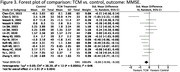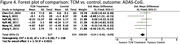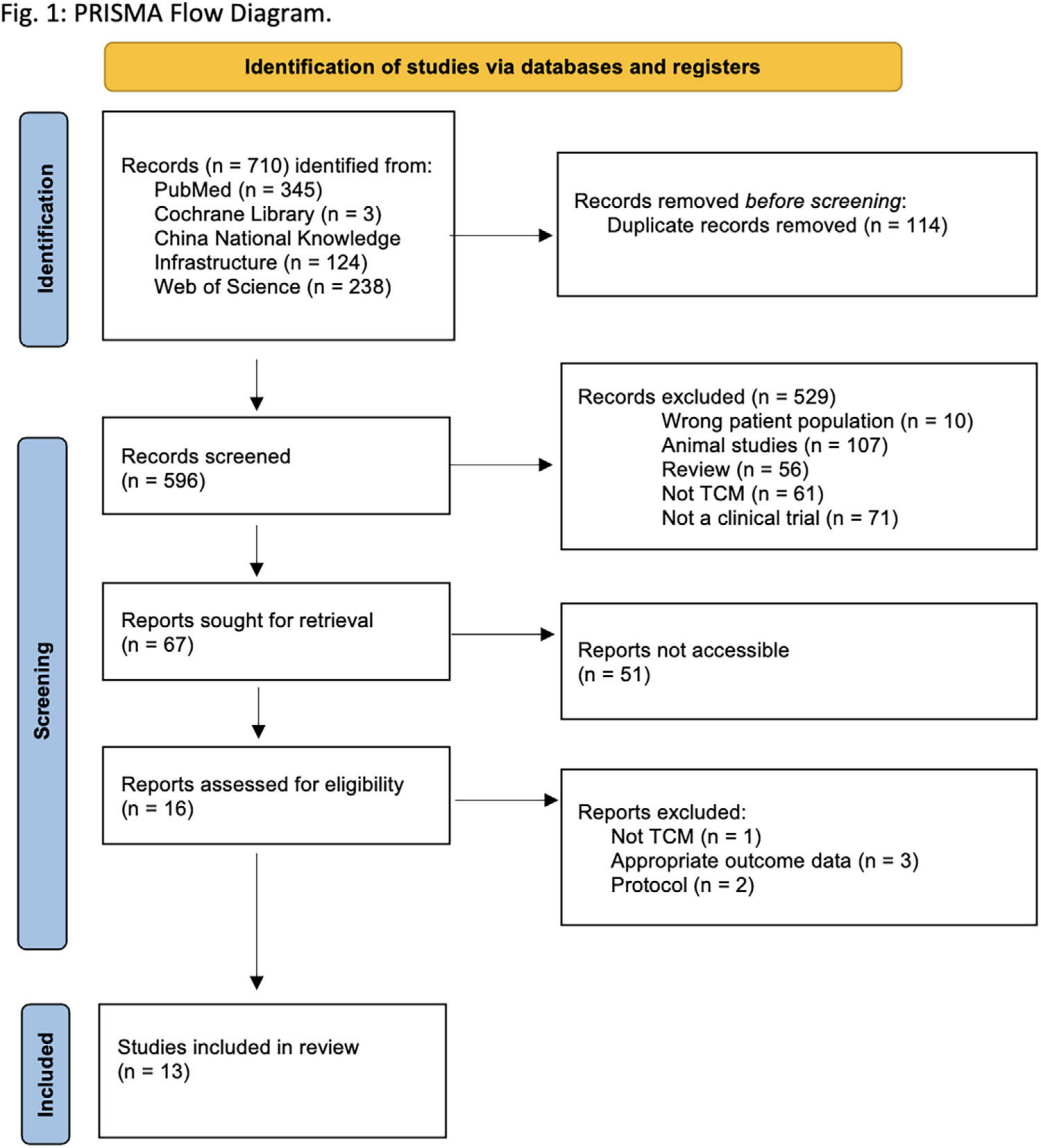# Traditional Chinese Medicine for Alzheimer’s Disease: A Systematic Review and Meta‐Analysis

**DOI:** 10.1002/alz.094963

**Published:** 2025-01-09

**Authors:** Yuqing Chen, Danning Zhang, Teng Chen, Lei Wen, Ruihua Hou

**Affiliations:** ^1^ School of Clinical Medicine, Addenbrooke’s Hospital, Cambridge United Kingdom; ^2^ Shandong University, Shandong Mental Health Center, Jinan, Shandong China; ^3^ Qilu Hospital of Shandong University, Jinan China; ^4^ Xiang’an Hospital, School of Medicine, Xiamen University, Xiamen, Fujian China; ^5^ University of Southampton, Southampton, Hampshire United Kingdom

## Abstract

**Background:**

The treatment of Alzheimer’s Disease (AD) remains a challenge for modern medicine due to its complex pathogenesis. Traditional Chinese Medicine (TCM) has accumulated significant value and success in prevention and treatment of diseases, and are regarded as new and promising candidates of AD pharmacological treatments. The current study aimed to systematically evaluate the current evidence base for TCM as a treatment for AD.

**Method:**

A systematic search of the literature was performed on electronic databases including PubMed, the Cochrane Library, Chinese National Knowledge Infrastructure (CNKI), and Web of Science. Thirteen studies were included in this review, subject to inclusion and exclusion criteria. Screening, data extraction, and quality assessment were undertaken following Preferred Reporting Items for Systematic Reviews and Meta‐Analyses guidelines. A total of 710 references were identified through searching the databases using the previously described search strategy. After excluding duplicates, titles and abstracts of 596 references were screened; of these, 13 were included in our systematic review and meta‐analysis, and a total of 1,183 participants were randomised. The primary outcome measures for efficacy were cognitive function, measured using the Standardized Mini‐Mental State Examination (MMSE) or ADAS‐cog. Secondary outcome measures included behavioural disturbances measured using the NPI, and activities of daily living assessed using the Alzheimer’s Disease Cooperative Study‐Activities of Daily Living (ADCS‐ADL).

**Result:**

Our results show a significant improvement of TCM compared to control for cognitive functioning, when measured on both the MMSE and ADAS‐CoG scales. This significant difference did not translate to scores of neuropsychiatric symptoms or ability of daily living activities. We show that TCM has potential to help to slow cognitive decline and improving cognitive function as compared to conventional drug treatment and placebo, but is limited in its effects on psychiatric symptoms and practical activities.

**Conclusion:**

These findings highlight TCM as potential adjuvant therapies, with conventional medicine, to improve effectiveness and reduce limitations of conventional AD drug regimes. Studies with larger sample size, rigorous study designs, accurate long‐term reporting, and correlation to neuropathological markers are needed in the future to enhance the evidence base for the use of TCM in AD patients, and to further confirm its efficacy.